# Range of motion of the mid-cervical spine: human versus goat

**DOI:** 10.1186/s13018-023-03896-1

**Published:** 2023-06-08

**Authors:** Jun Dong, Gelin Zhang, Yuan Sun, Xi Li, Xuyan Zhang, Min Liu, Ruoxi Liu, Liying Fan

**Affiliations:** Department of Orthopaedics, Second Affiliated Hospital of Xi’an Jiaotong University, No 157, West Five Road, Xincheng District, Xi’an, 710004 Shaanxi Province China

**Keywords:** Range of motion, Cervical spine, Human, Goat

## Abstract

**Background:**

The goat cervical spine represents a promising alternative for human specimen in spinal implant testing, but the range of motion (ROM) of the spine is lacking. We aimed to evaluate and compare the ROMs of fresh goat and human mid-cervical spine specimens.

**Methods:**

Ten fresh adult healthy male goat cervical spine specimens (G group) and ten fresh frozen adult healthy human cervical spine specimens (average age: 49.5 ± 12.1 years; 6 males, 4 females) (H group) were included. The ROMs of each specimen were biomechanically tested at the C_2–3_, C_3–4_, C_4–5_ and C_2–5_ levels at 1.5 Nm and 2.5 Nm torque and recorded. The ROMs of different levels of goat cervical samples were compared to those of human cervical samples using an independent sample *t* test. Significance was defined as a *P* value of less than 0.05.

**Results:**

At the C_2–3_, C_3–4_ and C_4–5_ levels, the ROMs of the goat cervical spine were significantly larger than those of the human cervical spine in all directions except extension under 1.5 Nm torque; under 2.5 Nm torque, the ROMs of the goat cervical spine at the C_2–3_ and C_3–4_ levels were significantly larger than those of humans in the pure movement of flexion, lateral bending and axial rotation, and the ROMs for axial rotation of the goat specimens and human specimens were comparable. Under both 1.5 Nm and 2.5 Nm torque, the goat cervical spine displayed a much greater ROM in all directions at the C_2–5_ level.

**Conclusions:**

Several segmental ROMs of fresh goat and human cervical spine specimens were recorded in this investigation. We recommend using goat cervical specimens as an alternative to fresh human cervical specimens in future studies when focusing only on the ROMs of C_2–3_, C_3–4_ and C_4–5_ in flexion under a torque of 1.5 Nm or the ROMs of C_2–3_ and C_3–4_ in flexion and rotation under a torque of 2.5 Nm.

**Supplementary Information:**

The online version contains supplementary material available at 10.1186/s13018-023-03896-1.

## Introduction

Cervical spondylosis is frequently treated surgically using spinal implants, such as plates, cages, screws and artificial discs [1–3]. In general, before being introduced into clinical use, these implants should go through several preclinical trials [4–6], such as an in vitro human cadaver biomechanical test and an in vivo animal experiment [7, 8]. However, due to the difficulty of acquiring fresh human cervical spine specimens and in vitro studies that failed to show changes in biomechanics, histology or functional behavior after employing implants [9–11], a large animal model imitating the human cervical spine is often used in trials [12–15]. The goat cervical spine is a suitable specimen for spinal implants among animal models due to its resemblance to humans in bone microstructure, content and remodeling [3, 10, 16, 17]. Many similarities exist between the vertebrae of goats and humans, although there are substantial differences in certain dimensions. Due to the differences in the macrostructure of the goat and human cervical spines, constructing an animal model requires a thorough understanding of the goat's biomechanical data, notably the range of motion (ROM). To date, there are not many methods for measuring the goat cervical spine, and those that have been employed mostly focus on functional spinal units [18]. However, there are a number of benefits to analyzing single motion segments, including simpler handling and a more accurate assessment of implant-related consequences. However, the entire cervical spine's ROM is limited. It is crucial to have a precise grasp of the ROM of the goat's lower cervical spine to carry out in vitro testing of spine implants. The purpose of the study was to evaluate and compare the ROMs of goat and human lower cervical spine specimens.

## Methods

### Specimens

This study was conducted according to the guidelines of the ethics committee of Xi’an Jiaotong University. Ten fresh human cervical specimens (C_2_–C_7_; average age: 49.5 ± 12.1 years; 6 males and 4 females; H group) were obtained by an informed donation from cadaveric material in accordance with state regulations from the Anatomy and Pathology Department of the Medical College of Xi'an Jiaotong University. Subjective examinations confirmed the absence of skeletal abnormalities. Digital X-ray films (QDR-2000; Hologic, Waltham, MA) were obtained to ensure that none of the specimens had osteoporosis. Ten fresh one-year-old goat cervical spine specimens (C_1_–C_7_; G group) were provided by the surgical experimental animal center of the Second Affiliated Hospital of Xi’an Jiaotong University. The muscle and soft tissue of the specimens were carefully removed, keeping the ligaments, capsules of facet joints and bony structures intact. The specimens were preserved at a temperature of − 20 °C.

### Biomechanical testing

**Fig. 1:**
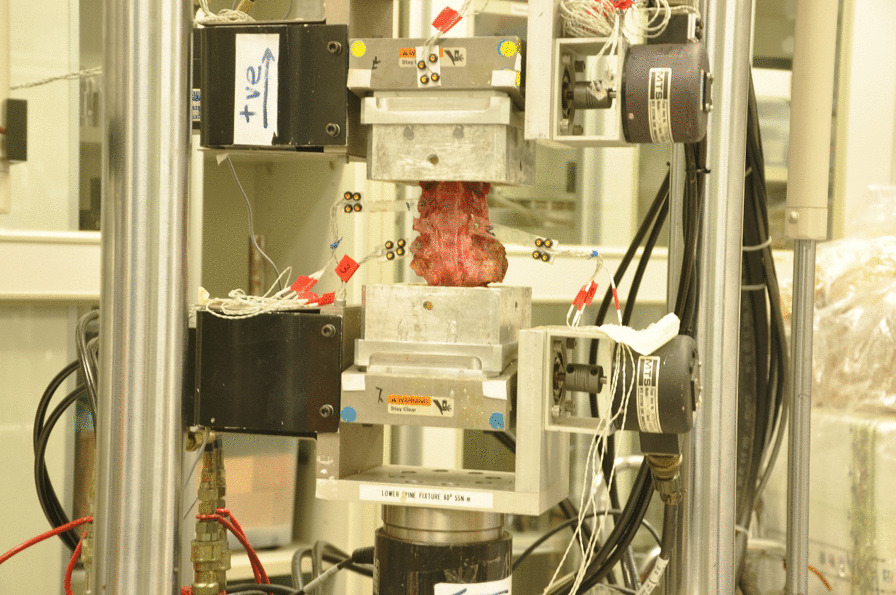
Photograph of a fresh human cervical specimen under biomechanical testing. The marker was attached to the top cylinder to prevent signal loss from the LEDs in the C_2_ vertebra

**Fig. 2:**
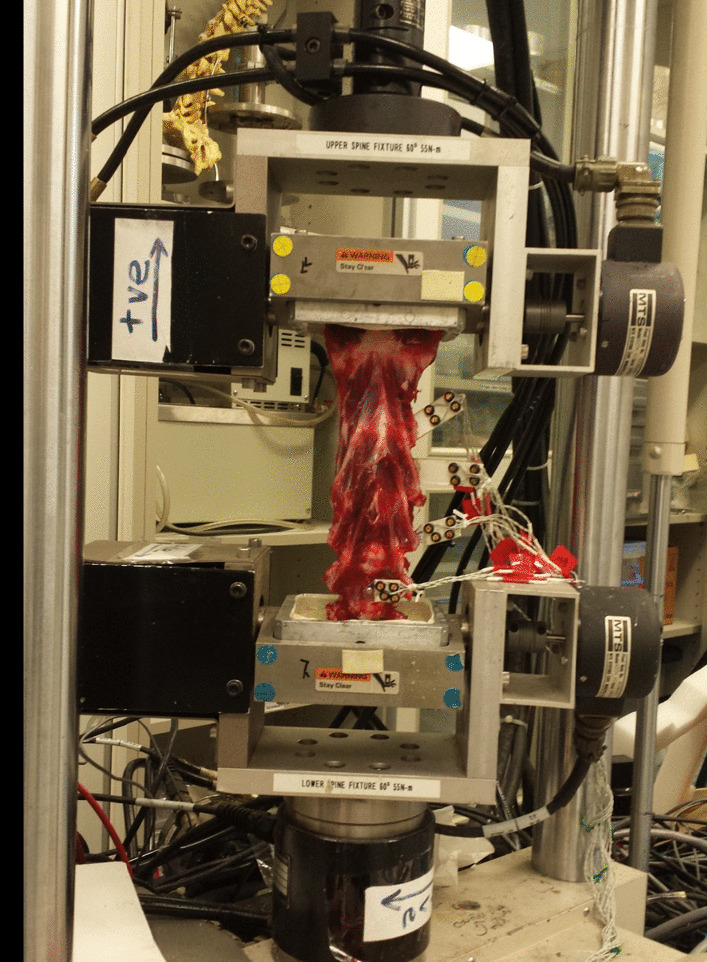
Photograph of a fresh goat cervical specimen under biomechanical testing

Biomechanical testing was completed in the Laboratory of Biomechanics, Department of Orthopaedics and Traumatology, Li Ka Shing Faculty of Medicine, The University of Hong Kong. Before testing, the specimens were thawed at room temperature for an hour and kept moist in plastic bags containing a small amount of 0.9% sodium chloride. The following steps were taken to enhance embedding: Specimens in the H group were initially fastened with several nails at C_2_ and C_7_; specimens in the G group fixed C_1_ and C_2_ as well as C_6_ and C_7_ together by nails. Afterward, the specimens were vertically embedded in two cylinders containing a mixture of N-(3-dimethylaminopropyl)-1,3-propylenediamine and bisphenol A-(epichlorohydrin) (1:1) (Figs. 1**, **2**)**.

Four light-emitting diodes (LEDs) were firmly attached to individual single flat plates, which were then attached to the C_2_, C_3_, C_4_ and C_5_ vertebrae. In total, 16 LEDs were used (Figs. 1**, **2). Ideally, the specimens would have an appropriate length; otherwise, capturing the instantaneous illumination of LEDs would be challenging. Because the specimens in the H group were too short, the first marker was placed on the upper mold rather than the C_2_ vertebral body. A pure moment was applied to the top end of the specimen using a material testing machine (MTS 858 Bionix machine, MTS System Inc., Minneapolis, MN, USA). Precision motion-capture equipment, an optoelectronic three-dimensional motion-capture system with three cameras (OPTOTRAK CERTUS, Northern Digital Inc., Waterloo, Canada), was used in the test.

In flexion–extension, left–right lateral bending and left–right axial rotation, a torque (1.5 Nm or 2.5 Nm) was applied to the upper end of the specimen. OPTOTRAK CERTUS (three-dimensional precision: 0.1 mm; measuring distinguishability: 0.01 mm, sampling frequency: 100 Hz) was used to record the instantaneous location of the LEDs in real time. A MATLAB program was used to convert these records to ROMs (MathWorks, Natick, MA, USA). Five loading cycles were performed in total, and the third cycle was used for statistical analysis.

### Statistical analysis

The data are represented as the mean ± SD. GraphPad Prism 5.0 (GraphPad Software, Inc.) was used to create the histogram. Statistical analyses were performed using SPSS 19.0 (SPSS Inc., Chicago, IL, USA). An independent sample t test was used to analyze the differences in ROM between the two groups. A p value of less than 0.05 was considered statistically significant.

## Results

### ROM under 1.5 Nm torque (Additional file 1: Table S1)

**Fig. 3:**
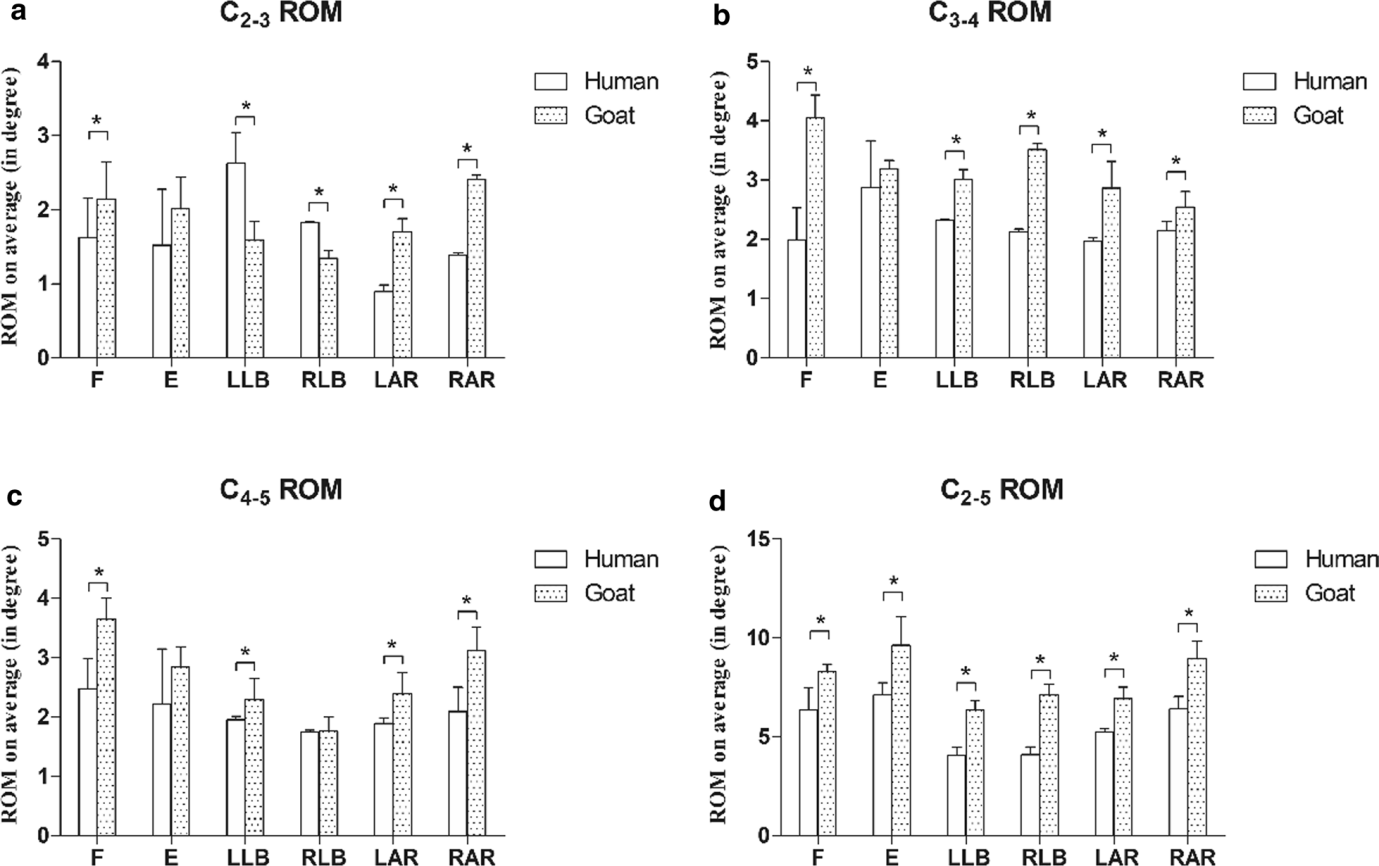
The fresh goat and human cervical spine specimens had ROMs of less than 1.5 Nm. **a** is for ROM in C_2–3_; **b** is for ROM in C_3–4_; **c** is for ROM in C_4–5_; and **d** is for ROM in C_2–5_. *f* flexion, *E* extension, *LLB* left lateral bending, *RLB* right lateral bending, *LAR* left axial rotation, *RAR *right axial rotation. *p* < 0.05 is denoted by an asterisk

Figure 3a shows the C_2–3_ ROM of the two groups of fresh cervical spine specimens under 1.5 Nm torque. The average C2-3 ROM of the human cervical spine specimens was 1.6 ± 0.5° of flexion, 1.5 ± 0.8° of extension, 2.6 ± 0.4° of left lateral bending, 1.8 ± 0.01° of right lateral bending, 0.9 ± 0.09° of left axial rotation and 1.4 ± 0.04° of right axial rotation **(**Fig. 3a). The average C2–3 ROM of the goat cervical spine specimens was 2.1 ± 0.5° of flexion, 2.0 ± 0.4° of extension, 1.6 ± 0.2° of left lateral bending, 1.3 ± 0.1° of right lateral bending, 1.7 ± 0.2° of left axial rotation and 2.4 ± 0.05° of right axial rotation **(**Fig. 3a). Significant differences in the ROM were observed between the two groups in flexion, lateral bending and axial rotation (*p* < 0.05) (Fig. 3a).

Figure 3b shows the C_3–4_ ROM of the two groups of fresh cervical spine specimens under 1.5 Nm torque. The average C3-4 ROM of the human cervical spine specimens was 2.0 ± 0.5° of flexion, 2.9 ± 0.8° of extension, 2.3 ± 0.02° of left lateral bending, 2.1 ± 0.05° of right lateral bending, 2.0 ± 0.06° of left axial rotation and 2.1 ± 0.2° of right axial rotation (Fig. 3b). The average C_3–4_ ROM of the goat cervical spine specimens was 4.0 ± 0.4° of flexion, 3.2 ± 0.1° of extension, 3.0 ± 0.2° of left lateral bending, 3.5 ± 0.1° of right lateral bending, 2.9 ± 0.5° of left axial rotation and 2.5 ± 0.3° of right axial rotation (Fig. 3b). Significant differences in ROM were observed between the two groups in all directions (*p* < 0.05) except extension (*p* > 0.05) (Fig. 3b).

Figure 3c shows the C_4–5_ ROM of the two groups of fresh cervical spine specimens under 1.5 Nm torque. The average C_4–5_ ROM of the human cervical spine specimens was 2.5 ± 0.5° of flexion, 2.2 ± 0.9° of extension, 2.0 ± 0.06° of left lateral bending, 1.8 ± 0.03° of right lateral bending, 1.9 ± 0.08° of left axial rotation and 2.1 ± 0.4° of right axial rotation (Fig. 3c). The average C_4–5_ ROM of the goat cervical spine specimens was 3.4 ± 0.5° of flexion, 2.8 ± 0.3° of extension, 2.3 ± 0.3° of left lateral bending, 1.8 ± 0.2° of right lateral bending, 2.4 ± 0.4° of left axial rotation and 3.1 ± 0.4° of right axial rotation (Fig. 3c). Significant differences in the ROM were observed between the two groups in flexion, left lateral bending and axial rotation (*p* < 0.05) (Fig. 3c).

Figure 3d shows the C_2–5_ ROM of the two groups of fresh cervical spine specimens under 1.5 Nm torque. The average C2–5 ROM of the human cervical spine specimens was 6.4 ± 1.1° of flexion, 7.1 ± 0.6° of extension, 4.1 ± 0.4° of left lateral bending, 4.1 ± 0.4° of right lateral bending, 5.2 ± 0.2° of left axial rotation and 6.4 ± 0.6° of right axial rotation (Fig. 3d). The average C2–5 ROM of the goat cervical spine specimens was 8.3 ± 0.4° of flexion, 9.6 ± 1.5° of extension, 6.3 ± 0.5° of left lateral bending, 7.1 ± 0.5° of right lateral bending, 6.9 ± 0.6° of left axial rotation and 9.0 ± 0.9° of right axial rotation (Fig. 3d). Significant differences in the ROM in all directions were observed between the two groups (*p* < 0.05) (Fig. 3d).

### ROM under 2.5 Nm torque (Additional file 2: Table S2)

**Fig. 4:**
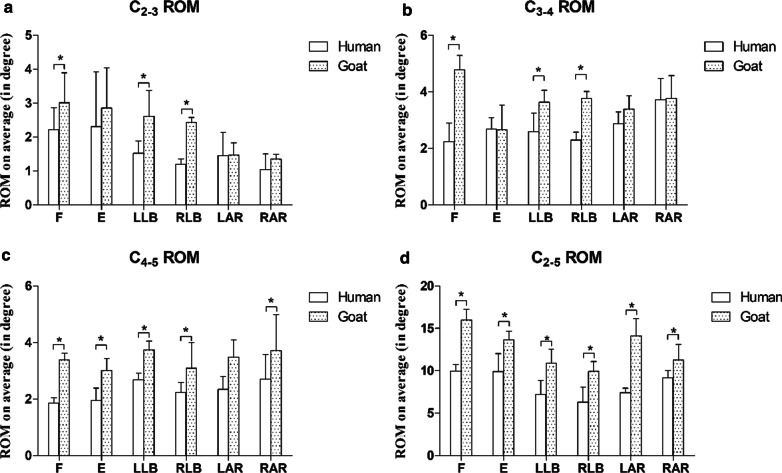
The fresh goat and human cervical spine specimens had ROMs of less than 2.5 Nm. **a** is for ROM in C_2–3_; **b** is for ROM in C_3–4_; **c** is for ROM in C_4–5_; and **d** is for ROM in C_2–5_. *F* flexion, *E* extension, *LLB* left lateral bending, *RLB* right lateral bending, *LAR* left axial rotation, *RAR* right axial rotation. *p* < 0.05 is denoted by an asterisk

The C2-3 ROMs of the two groups of fresh cervical spine specimens under 2.5 Nm torque are shown in Fig. 4a. The average C2–3 ROM of the human cervical spine specimens was 2.2 ± 0.6° of flexion, 2.3 ± 1.6° of extension, 1.5 ± 0.4° of left lateral bending, 1.2 ± 0.16° of right lateral bending, 1.5 ± 0.7° of left axial rotation and 1.0 ± 0.5° of right axial rotation (Fig. 4a). The average C2–3 ROM of the goat cervical spine specimens was 3.0 ± 0.9° of flexion, 2.9 ± 1.2° of extension, 2.6 ± 0.8° of left lateral bending, 1.3 ± 0.1° of right lateral bending, 1.5 ± 0.4° of left axial rotation and 1.3 ± 0.1° of right axial rotation (Fig. 4a). Significant differences were observed in the C2–3 ROM in flexion and lateral bending between the two groups (*p* < 0.05, *p* < 0.05) (Fig. 4a).

The C3-4 ROMs of the two groups of fresh cervical spine specimens under 2.5 Nm torque are shown in Fig. 4b. The average C3–4 ROM of the human cervical spine specimens was 2.2 ± 0.7° of flexion, 2.2 ± 0.7° of extension, 2.6 ± 0.6° of left lateral bending, 2.3 ± 0.3° of right lateral bending, 2.9 ± 0.4° of left axial rotation and 3.7 ± 0.8° of right axial rotation (Fig. 4b). The average C3–4 ROM of the goat cervical spine specimens was 4.8 ± 0.5° of flexion, 2.7 ± 0.9° of extension, 3.6 ± 0.5° of left lateral bending, 3.7 ± 0.2° of right lateral bending, 3.4 ± 0.5° of left axial rotation and 3.8 ± 0.8° of right axial rotation (Fig. 4b). Significant differences in the C3-4 ROM in flexion (*p* < 0.05) and lateral bending (*p* < 0.05) were observed between the two groups (Fig. 4b).

The C4–5 ROMs of the two groups of fresh cervical spine specimens under 2.5 Nm torque are shown in Fig. 4c. The average C4–5 ROM of the human cervical spine specimens was 1.9 ± 0.2° of flexion, 1.9 ± 0.4° of extension, 2.6 ± 0.2° of left lateral bending, 2.2 ± 0.4° of right lateral bending, 2.2 ± 0.4° of left axial rotation and 2.8 ± 0.9° of right axial rotation (Fig. 4c). The average C4–5 ROM of the goat cervical spine specimens was 3.4 ± 0.2° of flexion, 3.0 ± 0.4° of extension, 3.8 ± 0.3° of left lateral bending, 3.1 ± 0.9° of right lateral bending, 3.5 ± 0.6° of left axial rotation and 3.7 ± 1.3° of right axial rotation (Fig. 4c). Significant differences were observed between the two groups in the C_4–5_ ROM in all directions except left lateral bending (*p* > 0.05) (Fig. 4c).

The C2–5 ROMs of the two groups of fresh cervical spine specimens under 2.5 Nm torque are shown in Fig. 4d. The average C2–5 ROM of the human cervical spine specimens was 9.9 ± 0.8° of flexion, 9.9 ± 2.1° of extension, 7.2 ± 1.6° of left lateral bending, 6.3 ± 1.7° of right lateral bending, 7.4 ± 0.5° of left axial rotation and 9.2 ± 0.9° of right axial rotation (Fig. 4d). The average C2–5 ROM of the goat cervical spine specimens was 16.0 ± 1.3° of flexion, 13.6 ± 1.0° of extension, 10.9 ± 1.6° of left lateral bending, 9.9 ± 1.2° of right lateral bending, 14.1 ± 2.1° of left axial rotation and 11.3 ± 1.9° of right axial rotation (Fig. 4d). Significant differences in the C2–5 ROM in all directions were observed between the two groups in right lateral bending (*p* < 0.05) (Fig. 4d).

## Discussion

A variety of large animal species have been used as models for orthopedic research. Goat cervical spine specimens are frequently used as a promising substitute for human specimens in spinal implant testing due to their anatomical similarity to the fresh human cervical spine [18]. Several studies have reported the morphometrical information of the goat cervical spine [18–21]. However, there are few studies that contrast ROMs in goats and people. Based on the single-joint segment [22], Wilke et al. presented the biomechanical properties of a sheep model [22]. To find the ideal conditions for mimicking the characteristic features of the human cervical spine, our research examined the ROMs of goat specimens and compared them to those of people.

Figures 3 and 4 show the ROMs of the goat and human cervical spine specimens when subjected to 1.5 Nm and 2.5 Nm torque, respectively. The results revealed that the ROM of the goat cervical spine was significantly greater than that of the human cervical spine in all directions except extension at the C_2–3_, C_3–4_ and C_4–5_ levels under 1.5 Nm torque. The ROM of the goat cervical spine at the C_2–3_ and C_3–4_ levels was significantly greater than that of the human cervical spine in pure flexion and lateral bending under a torque of 2.5 Nm and was comparable in axial rotation. In all directions except left axial rotation, the C_4–5_ ROM of the goat specimens was significantly larger than that of the human specimens. Under both 1.5 Nm and 2.5 Nm of torque, the goat cervical spine showed significantly more ROM in all directions at the C_2–5_ level. The ROMs of the specimens subjected to 2.5 Nm torque were greater than those of the specimens subjected to 1.5 Nm torque. As the ROM of the goat cervical spine is generally larger than that of the human cervical spine, goat cervical spines may not be the best alternative to human cervical specimens. However, considering the rarity of fresh human cervical spine specimens, fresh goat cervical spine specimens may still be used in the clinic. The ROMs of the goat cervical specimens at 1.5 Nm and 2.5 Nm torque showed some similarity to those of human specimens at certain levels. This finding suggests that goat cervical spine specimens can still be an alternative to fresh human cervical spine specimens only when the study focuses on the ROMs of C_2–3_, C_3–4_ and C_4–5_ in flexion at 1.5 Nm torque, as well as C_2–3_ and C_3–4_ in flexion and axial rotation at 2.5 Nm torque. Similar findings were obtained in another study by DeVries et al., who discovered that the flexion ROM was greater than the extension ROM [21]. However, the axial rotation and lateral bending values in DeVries’s study were the sums of the right and left sides, while the ROMs in our study were recorded in six independent directions. The ROM on the left should, in theory, be equal to the ROM on the right. During testing, however, only a few symmetrical ROMs were observed. A possible reason is that it was difficult to find the neutral position of the specimens, and as a result, the specimens may have been tilted. In our study, the ROMs of goats under 2.5 Nm were lower than those reported by DeVries et al. This might be explained by the factors mentioned above. The change in segmental ROMs should be evident, especially as the OPTOTRAK CERTUS is highly accurate. As the goat cervical spine does not carry the same axial stress as the human cervical spine, axial preload was not studied in this experiment. In this study, two kinds of pure loads (1.5 Nm and 2.5 Nm) were applied to the upper end (C_2_) of the embedded specimen, and the lower end of the specimen (C_5_) remained fastened to the cylinder of the MTS machine. We also observed increased segmental ROMs in goat and human specimens from the upper to the lower level in flexion, left and right bending, and left and right axial rotation; these progressions were more noticeable in flexion and axial rotation. These findings suggest that the most flexible parts of the cervical spine are in the center, which may explain why the C_4–5_ intervertebral disc is prone to degeneration [23–25].

This study described the ROMs of the goat cervical spine, which is routinely employed in in vitro testing of spine implants. These findings may aid in determining the suitability of the goat cervical spine as a model for spinal research.

This study has limitations. First, the goat cervical spine specimens were taken from one-year-old goats, which means these ROMs are representative of a particular age. Second, the reported ROMs in our study were just from C_2_ to C_5_, while C_1–2_, C_5–6_ and C_6–7_ were not measured due to the shortage of LEDs. The development of the cervical vertebrae of goats of different ages and sexes may vary to some extent, and there may be some differences in mobility due to the different degrees of degeneration that may exist in goats of different ages. Although the goats selected in our experiments were all 1-year-old male goats, we still cannot guarantee that the mobility of the cervical vertebrae of all the goats in the experiments was consistent; the elimination of muscles and soft tissues could not be completely consistent, so the residual soft tissues of the cervical vertebrae of the goats would have a certain degree of influence.

## Conclusion

In this study, multiple segmental ROMs of fresh goat and human cervical spine specimens were presented. The results might aid with in vitro spinal implant biomechanical testing. At the same applied moment, goat specimens demonstrated a greater ROM than human specimens in general. A moment of 1.5 Nm is quite small and does not take into consideration the physiological burden experienced by people. In light of this, using human samples is advised or suggested, although using goat samples is a potential but constrained substitute for cervical spine implant testing.

## Supplementary Information

**Additional file 1**. ROM of the human and goat fresh cervical spine specimens under 1.5 Nm torque.

**Additional file 2**. ROM of the human and goat fresh cervical spine specimens under 2.5 Nm torque.

## Data Availability

All authors state that all data and materials as well as software application or custom code can support our published claims and comply with field standards.
