# Supplementary material for: Range of motion of the mid-cervical spine: human versus goat
Source: J Orthop Surg Res. 2023 Jun 8;18:416. doi: 10.1186/s13018-023-03896-1 (PMC10249303; doi:10.1186/s13018-023-03896-1)
Supplement: Supplementary file 1 — Additional file 1. ROM of the human and goat fresh cervical spine specimens under 1.5 Nm torque. [file 13018_2023_3896_MOESM1_ESM.docx]

Table 1. ROM of the human and goat fresh cervical spine specimens under 1.5 Nm torque.

|  | F | | E | | LLB | | RLB | | LAR | | RAR | |
| --- | --- | --- | --- | --- | --- | --- | --- | --- | --- | --- | --- | --- |
|  | H | G | H | G | H | G | H | G | H | G | H | G |
| C_2-3_ | 1.6±0.5 | 2.1±0.5 | 1.5±0.8 | 2.0±0.4 | 2.6±0.4 | 1.6±0.2 | 1.8±0.01 | 1.3±0.1 | 0.9±0.09 | 1.7±0.2 | 1.4±0.04 | 2.4±0.05 |
| C_3-4_ | 2.0±0.5 | 4.0±0.4 | 2.9±0.8 | 3.2±0.1 | 2.3±0.02 | 3.0±0.2 | 2.1±0.05 | 3.5±0.1 | 2.0±0.06 | 2.9±0.5 | 2.1±0.2 | 2.5±0.3 |
| C_4-5_ | 2.5±0.5 | 3.4±0.5 | 2.2±0.9 | 2.8±0.3 | 2.0±0.06 | 2.3±0.3 | 1.8±0.03 | 1.8±0.2 | 1.9±0.08 | 2.4±0.4 | 2.1±0.4 | 3.1±0.4 |
| C_2-5_ | 6.4±1.1 | 8.3±0.4 | 7.1±0.6 | 9.6±1.5 | 4.1±0.4 | 6.3±0.5 | 4.1±0.4 | 7.1±0.5 | 5.2±0.2 | 6.9±0.6 | 6.4±0.6 | 9.0±0.9 |

Note: F, flexion; E: extension; LLB: left lateral bending; RLB: right lateral bending; LAR: left axial rotation; RAR: right axial rotation; H: human; G: goat.
