# Supplementary material for: Range of motion of the mid-cervical spine: human versus goat
Source: J Orthop Surg Res. 2023 Jun 8;18:416. doi: 10.1186/s13018-023-03896-1 (PMC10249303; doi:10.1186/s13018-023-03896-1)
Supplement: Supplementary file 2 — Additional file 2. ROM of the human and goat fresh cervical spine specimens under 2.5 Nm torque. [file 13018_2023_3896_MOESM2_ESM.docx]

Table 2. Table 1. ROM of the human and goat fresh cervical spine specimens under 2.5 Nm torque.

|  | F | | E | | LLB | | RLB | | LAR | | RAR | |
| --- | --- | --- | --- | --- | --- | --- | --- | --- | --- | --- | --- | --- |
|  | H | G | H | G | H | G | H | G | H | G | H | G |
| C_2-3_ | 2.2±0.6 | 3.0±0.9 | 2.3±1.6 | 2.9±1.2 | 1.5±0.4 | 2.6±0.8 | 1.2±0.16 | 1.3±0.1 | 1.5±0.7 | 1.5±0.4 | 1.0±0.5 | 1.3±0.1 |
| C_3-4_ | 2.2±0.7 | 4.8±0.5 | 2.2±0.7 | 2.7±0.9 | 2.6±0.6 | 3.6±0.5 | 2.3±0.3 | 3.7±0.2 | 2.9±0.4 | 3.4±0.5 | 3.7±0.8 | 3.8±0.8 |
| C_4-5_ | 1.9±0.2 | 3.4±0.2 | 1.9±0.4 | 3.0±0.4 | 2.6±0.2 | 3.8±0.3 | 2.2±0.4 | 3.1±0.9 | 2.2±0.4 | 3.5±0.6 | 2.8±0.9 | 3.7±1.3 |
| C_2-5_ | 9.9±0.8 | 16.0±1.3 | 9.9±2.1 | 13.6±1.0 | 7.2±1.6 | 10.9±1.6 | 6.3±1.7 | 9.9±1.2 | 7.4±0.5 | 14.1±2.1 | 9.2±0.9 | 11.3±1.9 |

Note: F, flexion; E: extension; LLB: Left lateral bending; RLB: Right lateral bending; LAR: left axial rotation; RAR: right axial rotation; H: human; G: goat.
